# Longitudinal Evaluation of an Integrated Post–COVID-19/Long COVID Management Program Consisting of Digital Interventions and Personal Support: Randomized Controlled Trial

**DOI:** 10.2196/49342

**Published:** 2023-10-04

**Authors:** Christina Derksen, Robin Rinn, Lingling Gao, Alina Dahmen, Cay Cordes, Carina Kolb, Petra Becker, Sonia Lippke

**Affiliations:** 1 Health Psychology and Behavioural Medicine, Constructor University Bremen Bremen Germany; 2 Wolfson Institute of Population Health, Queen Mary University of London London United Kingdom; 3 Dr. Becker Kiliani-Klinik, Dr. Becker Klinikgruppe Bad Windsheim Germany; 4 Dr. Becker Klinikgruppe Cologne Germany

**Keywords:** postacute COVID-19 syndrome, PACS, symptom reduction, work ability, social participation, personal pilots, digital interventions, empowerment, randomized controlled trial, propensity score matching, COVID-19

## Abstract

**Background:**

The postacute COVID-19 syndrome (PACS) can be addressed with multidisciplinary approaches, including professional support and digital interventions.

**Objective:**

This research aimed to test whether patients who received a health care facilitation program including medical internet support from human personal pilots and digital interventions (intervention group [IG] and active control group [ACG]) would experience fewer symptoms and have higher work ability and social participation than an untreated comparison group (CompG). The second objective was to compare the impact of a diagnostic assessment and digital interventions tailored to patients’ personal capacity (IG) with that of only personal support and digital interventions targeting the main symptoms (ACG).

**Methods:**

In total, 1020 patients with PACS were recruited. Using a randomized controlled trial design between the IG and the ACG, as well as propensity score matching to include the CompG, analyses were run with logistic regression and hierarchical-linear models.

**Results:**

Symptoms decreased significantly in all groups over time (βT1-T2=0.13, *t*_549_=5.67, *P*<.001; βT2-T4=0.06, *t*_549_=2.83, *P*=.01), with a main effect of the group (β=–.15, *t*_549_=–2.65, *P*=.01) and a more pronounced effect in the IG and ACG compared to the CompG (between groups: βT1-T2=0.14, *t*_549_=4.31, *P*<.001; βT2-T4=0.14, *t*_549_=4.57, *P*<.001). Work ability and social participation were lower in the CompG, but there was no significant interaction effect. There were no group differences between the IG and the ACG.

**Conclusions:**

Empowerment through personal pilots and digital interventions reduces symptoms but does not increase work ability and social participation. More longitudinal research is needed to evaluate the effects of a diagnostic assessment. Social support and digital interventions should be incorporated to facilitate health care interventions for PACS.

**Trial Registration:**

ClinicalTrials.gov NCT05238415; https://classic.clinicaltrials.gov/ct2/show/NCT05238415.

**International Registered Report Identifier (IRRID):**

RR2-10.1186/s12879-022-07584-z

## Introduction

### Background

According to the World Health Organization (WHO), worldwide, approximately 769 million people have contracted SARS-CoV-2, as detected by a positive polymerase chain reaction (PCR) test as of August 2023 [1]. Although a vast majority of these patients (99.9%) survived their infection, 7.5%-41.0% of nonhospitalized patients suffer from long-term health impairments. Risk factors include a higher age, the female sex, and previous health conditions [2,3]. The symptoms can be classified as long COVID if they persist for more than 4 weeks after an acute infection or post–COVID-19 if they persist for more than 12 weeks [4]. Both terms can be summarized under the name “postacute COVID-19 syndrome” (PACS). Although guidelines to treat PACS exist [5], there is no generally accepted diagnostic method (eg, [6]).

One reason that there is no accepted diagnostic method is that various diverse psychological and physiological symptoms may fall under the potential diagnosis of PACS (see Ref. [7] for an overview of the symptoms). The most common symptoms include fatigue, muscle or joint pain, cough and shortness of breath, altered smell and taste, and (neuro)psychological symptoms, such as concentration problems, impaired quality of sleep, and anxiety [8]. It has also been difficult to design a generic diagnostic instrument for PACS because its pathophysiological mechanisms are rather unknown. There is evidence that PACS is driven by several organ-specific pathophysiological mechanisms, such as tissue damage and inflammatory reactions caused by autoimmune reactions or viral persistence [9,10]. This medical complexity, the diversity of symptoms, and the limitation of the functional capacities of individuals make it difficult to determine which symptoms were caused by PACS, consequently leading to either insufficient diagnostic testing or overuse of diagnostic assessment [11].

To offer clear guidelines and thus address the challenge of diagnosing PACS, several authors have proposed diagnostic criteria. These include criteria regarding symptoms and clinical features for symptomatic and asymptomatic acute SARS-CoV-2 infections that could not be attributed to another cause, as well as duration criteria for persisting symptoms [3,12]. However, research using standardized methods to diagnose post–COVID-19/long COVID is sparse and has not been implemented widely into care. Thus, health care professionals, such as primary care workers, face several uncertainties. PACS, as a relatively new syndrome, is missing evidence-based research and is surrounded by a high amount of misinformation [13-15]. These uncertainties are likely to detain health care professionals from effectively guiding the diagnostic and treatment process [16]. Consequently, PACS is not diagnosed and treated effectively, leading to a risk of chronification and worsening of patient conditions, ultimately negatively affecting individuals’ functional status [17].

In line with the difficulties regarding diagnostics, PACS treatment is also fragmented depending on symptom expression. Currently, individuals are treated mostly in primary care for specific symptoms. Instead, a more holistic approach, such as the inclusion of multiprofessional teams of health care specialists, is needed to target PACS. However, it is difficult for patients to navigate through the health care system and find appropriate treatment options [14,18]. Struggling with symptoms, especially in the case of neuropsychological impairments (eg, brain fog, concentration problems, and fatigue), can lead to feelings of isolation, difficulties in engaging in activities of daily living, and loss of work ability [19]. Thus, PACS is related to disruptions of social participation, long absence from work, and impaired work ability [2], and more in-person or online support is required. A personal counselor could provide such support, but this has not been tested in a structured way.

In an international cohort, Davis et al [7] found that 7 months after their acute infection, only 27.3% of all individuals who were recruited from long COVID support groups resumed working their normal hours and that 22.3% of patients did not return to work. Although this sample was highly selective due to its recruitment, the study illustrates the impact of PACS on individuals’ work ability. It becomes evident that PACS burdens not only the individual and the health care system (ie, through a prolonged treatment process) but also the economy and overall society [20], increasing the need for effective care and treatment options.

To address these long-term negative effects of PACS, researchers have recommended multidisciplinary approaches, such as (tele)rehabilitation, digital, medical internet, and eHealth interventions targeting social support [21-23]. However, Schrimpf et al [16] found that general practitioners (GPs) rated current treatment options as “poor,” which shows that promising approaches have rarely been applied in general practice. Similarly, only few PACS treatment programs have been validated, especially those using digital elements [24]. This is a huge shortcoming as medical treatments and self-management programs play an important role in the management of PACS [13,25]. Research has shown that digital interventions with partly asynchronous and partly face-to-face elements can improve patients’ health status [21,26-28].

Telerehabilitation programs could improve physical capacity and the quality of life by reducing dyspnea and fatigue symptoms [29]. Furthermore, in their “Hope Program for Long COVID,” Wright et al [30] introduced a cocreative self-management intervention focusing on digital peer support and goal setting that was acceptable and effective in increasing self-efficacy and mental well-being, which are important factors in recovery. Despite good evidence that digital approaches targeting self-management of PACS can be effective, the programs that address PACS symptoms have rarely been evaluated. This includes their their long-term effectiveness in symptom reduction and work ability, especially in controlled research designs with a digital intervention platform augmented by human personal support and tailoring based on diagnostic assessments. Therefore, the primary objective of this study was to address this gap in the literature.

### Study Hypotheses

To overcome gaps in the literature, this study was conducted within the “ASAP—Assisted Immediate Augmented Post-/Long-COVID Plan” project [31]. We aimed to develop and evaluate an interdisciplinary health care facilitation program based on professional personal pilot support and digital interventions, both delivered as medical internet aid. The project in which the data for the study were collected included (1) online screening to diagnose PACS, (2) a human personal pilot concept that guided patients to recover, (3) a diagnostic assessment, and (4) digital interventions based on assessment results or the most prevalent symptoms (digital medical rehabilitation platform with physical exercises, mindfulness training, and sensory and functional training).

Overall, the project aimed to increase the self-management of patients with PACS to ensure their empowerment regarding their health care facilitation. We assumed that the interventions would be more effective than spontaneous remission (comparison group [CompG]). To account for the effect of a thorough diagnostic assessment, 1 group received all interventions (intervention group [IG]) and 1 group did not receive a diagnostic assessment but received all personal support and digital interventions (active control group [ACG]). We assumed that the diagnostic assessment would help inform subsequent personal support and digital interventions, thus increasing the effectiveness of the program. Hence, we hypothesized that:

- Hypothesis 1: Patients who receive support from personal pilots and digital interventions (IG and ACG) experience fewer symptoms after the program and have higher work ability and social participation than the untreated CompG.
- Hypothesis 2: For patients who receive a thorough diagnostic assessment and accordingly tailored digital interventions based on their personal capacity (IG), improvements regarding symptom reduction, social participation, and work ability will be larger than for patients who only receive the personal pilot and digital interventions targeting their main symptoms (ACG).
- Hypothesis 3: As previous research has shown that symptoms can negatively affect patients regarding their work ability, we hypothesized that symptom reduction over the course of the pandemic will be associated with higher work ability and higher social participation after the program.

## Methods

### Study Design

This partially randomized controlled trial, which followed the Consolidated Standards of Reporting Trials (CONSORT) guidelines (Multimedia Appendix 1), is part of the German project ASAP funded by the Bavarian State Office for Health and Food Safety [32]. The study was registered at ClinicalTrials (ClinicalTrials ID: NCT05238415), and details have been published in the study protocol [31].

### Ethical Considerations

Ethical approval was obtained from the Constructor (formerly Jacobs) University Bremen Ethics Committee (application no: 2021_08). We ensured that all participants read the study information and provided informed consent before data collection. All data were collected and matched using pseudonymous participant codes.

### Recruitment

In this partially randomized controlled trial, 3 groups of patients were recruited, including an IG, an ACG, and a care-as-usual CompG (which did not receive any specific treatment). All individuals who were interested in taking part in the study participated in an initial online screening (time point T1) to identify whether they had PACS based on the following criteria (see Refs. [31,33]) and to make sure that potential participants matched the inclusion and exclusion criteria:

- The participants had ≥3 (of 14) PACS symptoms with severity ≥2 on a scale from 0 (no problem) to 3 (extreme problem).
- Symptoms could not be attributed to other causes by the participants.
- Symptoms were new or exacerbated after a SARS-CoV-2 infection.
- The SARS-CoV-2 infection had been diagnosed more than 4 weeks ago.
- The participants had not fully or substantially recovered from their PACS symptoms.
- The participants suffered from impairments in their daily life.

Additional inclusion criteria were that participants were between 18 and 60 years old, had not received previous treatment (eg, medical rehabilitation), and did not work in the health care sector, as that would have made them eligible for specialized treatment in Germany. Lastly, participants had no or only a low “care degree.” In Germany, a care degree indicates to what extent a person is in need of care: A care degree of 1 describes “slight impairment of independence” of those in need of care and guarantees them corresponding benefits from long-term care insurance. With a care degree of 5, long-term care insurance funds certify that the insured persons have the “most severe impairment of independence, with special requirements for nursing care” and thus approve the most extensive benefits from the long-term care fund.

The IG and ACG were recruited via a separate online link exclusively in Bavaria through press releases, social media campaigns, and GPs who received flyers via mail and were asked to forward the information to their patients if they thought they might have PACS. After reading the study information and providing consent to participate in the study, participants started with the initial screening. If the screening was positive, they were then admitted to the study program after randomization in a 1:2 ratio by the rolling of a die. Patients with a 1 or 2 on the die were allocated to the IG, while patients for whom the die showed 3-6 were randomized to the ACG. In some cases, randomization was not possible (eg, if patients could not attend in-person interventions due to organizational reasons or were recruited after the assessment dates were booked). Thus, there was an exception for 30 (13.3%) patients who were reallocated to the ACG.

### Study Process

Patients were not blinded regarding the study groups in order to ensure full informed consent. After the screening and randomization, both the IG and the ACG were contacted by trained persons working in the project, who were called “personal pilots.” The pilots evaluated the inclusion criteria in a first contact over the phone. This first contact was standardized to assess PACS symptoms, the timeline of the infection and development of PACS symptoms, potential previous treatment and management of the condition, and age, residency, and current employment. If any concerns arose regarding the eligibility of the patient, the personal pilots clarified with the research team and the patient. The pilots also provided more information about the study process during the first contact and organized the participation in the project. After the initial contact, weekly telephone meetings were arranged, and the patients received an introduction to the digital intervention platform over 2 weeks. They were asked to follow an unspecific treatment plan including information about hand hygiene and general health behaviors, such as a healthy diet. After that, patients were asked to answer the second questionnaire regarding their current symptoms, social participation, and work ability (T2).

In addition to synchronous telephone contacts with the personal pilots and the T2 questionnaire, the IG was administered a 3-day diagnostic assessment in the cooperating specialized neurological stationary rehabilitation clinic. After the assessment, patients in the IG answered another short questionnaire (T3) on their symptoms and received asynchronous digital interventions over 6 weeks that were tailored to their cognitive and physical capacities determined in the assessment.

The ACG received no assessment in the clinic but received synchronous telephone contacts and asynchronous digital interventions based on their main symptoms (ie, fatigue, cognitive impairment, or cardiorespiratory symptoms). During the 6 weeks, the personal pilots had biweekly meetings with the patients to evaluate the patients’ progress and help with technical issues. They were also available on demand. After the digital interventions, the patients answered another questionnaire (T4) regarding symptoms, social participation, and work ability. The IG and ACG answered a last follow-up questionnaire (T5) 6 weeks after finishing the digital interventions.

Individuals in the CompG who answered not only the baseline questionnaires but also the repeated measures were included in the study. They were recruited online not only in Bavaria but in the entire country of Germany through social media campaigns and press releases. After providing informed consent, individuals took part in the screening (T1) and were invited to provide their email addresses for the follow-up questionnaires after 4 (T2) and 10-12 weeks (comparable to T4 in the IG/ACG). The email addresses were not matched to any other data to ensure anonymity.

**Figure 1:**
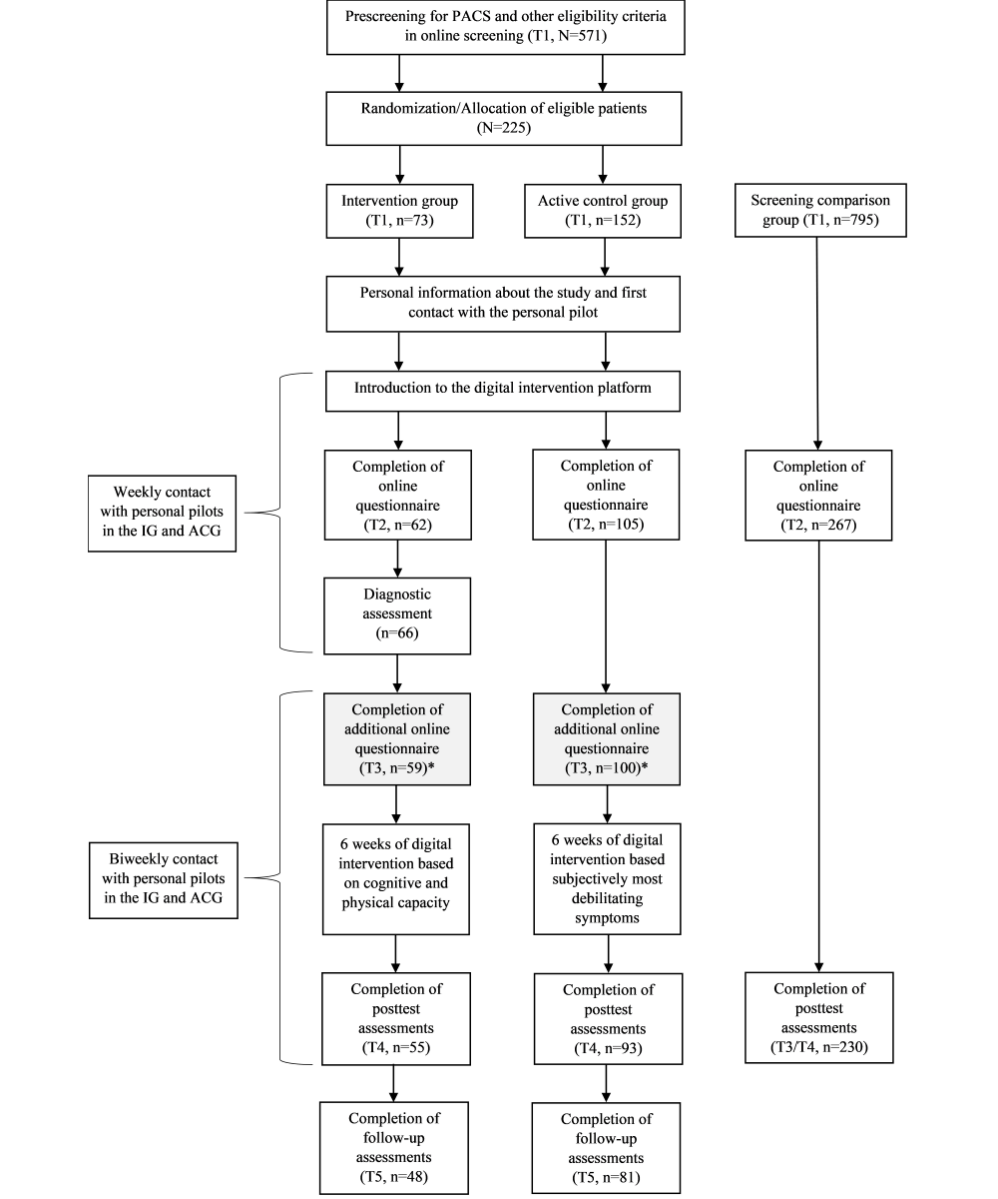
Flowchart of the study design and participant dropout. *The T3 questionnaire was a short measure capturing symptom severity directly after the assessment in the IG and a comparable time point in the ACG. Data from T3 was not analyzed in this study. In the CompG, T3 was comparable to T4 in the IG and ACG and was thus named T3/T4 in this manuscript. ACG: active control group; IG: intervention group; PACS: postacute COVID-19 syndrome.

Figure 1 includes a flowchart of the study design and participant dropout.

### Interventions

Patients in the IG participated in the diagnostic assessment in the clinic, and their digital interventions were tailored based on their personal capacity identified in the assessment. Patients in the ACG did not take part in the assessment but received digital interventions based on their main symptoms. Both the IG and the ACG received support from their personal pilots. The CompG only responded to the online questionnaires and did not receive any intervention.

#### Diagnostic Assessment

The IG was invited to a 3-day inpatient assessment at a specialized neurological stationary rehabilitation clinic. An interdisciplinary team from the fields of neurology, cardiology, and pulmonology carried out a detailed medical history and examination (clinical and technical). These were supplemented by tests from physiotherapy, occupational therapy, and neuropsychology to assess the functional status and limitations that indicated the presence of PACS. All tests were based on the recommendations of the “S1-Guideline on Long/Post-COVID” [5]. On the first day, a thorough medical review was conducted to guide subsequent tests (see Table A1 in Multimedia Appendix 2). The assessment aimed to result in a diagnosis for PACS by the clinicians and to determine the need for further diagnostics and treatment options (eg, further neurological tests or a rehabilitation measure as well as an assessment of the overall capacity for subsequent digital interventions).

#### Digital Interventions

After enrollment and the first contact with their personal pilots, patients received access to the digital platform. Initially, patients from the IG and ACG familiarized themselves with the platform using unspecific interventions, such as general information about hygiene, nutrition, and exercise. In the ACG, the personal pilots decided together with the patients which symptom-specific plan would be the most fitting. They could choose between digital interventions focusing on fatigue, neurocognitive symptoms, or cardiorespiratory symptoms. Patients in the IG received digital interventions based on their physiological and cognitive capacity that was determined during the assessment in the clinic. Digital intervention plans were developed specifically targeted for low, medium, and high capacity. Capacity was assessed both subjectively and objectively and finally summarized by a physician. In the online screening, patients subjectively reported how strongly their condition affected their resilience and capacity to cope with everyday life demands. This information was explored in the admission interview for the diagnostic assessment by medical staff. To include more objective methods, typical parameters for echocardiography (performance of the heart at rest), ergometry, and spirometry (lung function) were assessed to determine the cardiopulmonary exercise capacity. Impairments regarding patients’ neuropsychological capacity were assessed with comprehensive testing of cognitive abilities (attention, thinking, and memory). In a physiotherapeutic and occupational therapy assessment, individual tests were performed and the accuracy as well as the stamina in this assessment were evaluated. In the final medical interview and the overall medical assessment, an assessment of exercise capacity was thus made in the areas of cardiology and pulmonology, neurology, psychosomatics, and neuropsychology. If reduced resilience was determined in one area, then remedial therapy was recommended in the treatment plan for this target. There were no cutoff values for the integration of capacity measures, as the aim was to use the patients’ own subjective assessments and the more objective diagnostic assessments as a basis to categorize the patients’ capacity as low, medium, or high for the digital interventions.

Contents of the digital interventions included breathing and relaxation exercises, mindfulness training, meditation, physical strength tasks, and sensory as well as functional training. All patients in the IG and ACG received personal guidance from their personal pilots and were encouraged to adapt the exercises individually, when needed. For this purpose, based on physiotherapeutic expertise, an exercise manual with different variants of the physical exercises was provided. The focus of the digital training was to strengthen the patients’ self-management. Patients took part individually in the asynchronous offers and were expected to adhere to the daily intervention plan. The time spent on the daily activities on average was 50.4 minutes (range 44.6-58.7, depending on the level of intensity) in the IG and 41.9 minutes (range 32.5-48.7) in the ACG.

#### Human Support and Personal Pilots

Trained professionals functioning as personal pilots had 2 main functions. First, they guided patients through the study process in a mainly synchronous but partially also asynchronous way over the phone and via email. Each patient was assigned a personal pilot who was available throughout their study participation. The pilots reminded the patients of the questionnaires, arranged the inpatient assessment in the IG, and provided recommendations and help for the asynchronous digital interventions.

Second, the pilots empowered the patients in their individual approaches to cope with their PACS to enable them in finding the best-possible treatment. To achieve this, the pilots contacted all patients weekly in the beginning and biweekly during the digital interventions to reflect on the process, review the patients’ needs, and work together on options for future care. The pilots also motivated the patients to identify and access further diagnostic and therapy options, as well as engage in activities of daily living, while pacing themselves to foster recovery. All pilots were either qualified psychologists, health scientists, or health care professionals (eg, physiotherapists) and were trained in motivational interviewing [34].

### Measures

To evaluate the ASAP project, the IG and ACG were compared with each other and with the CompG over time regarding primary and secondary outcomes. Outcomes included symptom severity (assessed at all time points), work ability (measured at T2, T4, and T5), and social participation (measured at T2, T4, and T5).

*Symptom severity* was measured at all time points with a scale developed for the project [31]. In the screening and subsequent questionnaires, a list of 14 common PACS symptoms (eg, fatigue, muscle pain, respiratory problems) were listed. Patients indicated whether they suffered from the symptoms on a scale from 0 (no problem) to 3 (extreme problem). The sum of scores was calculated. Cronbach α was .80 at T1 across groups.

*Work ability* was measured with a single item from the Work Ability Index (WAI) [35], namely “If you give your best-ever work ability a score of 10, how many points would you give your current work ability?” The answer options ranged from 0 (completely unable to work) to 10 (currently best work ability).

*Social participation* was measured using a 12-item questionnaire [36] that assessed participants’ ability to engage in specific activities (eg, taking care of one’s health, eating healthy, exercising, or taking medication). Answer options ranged from 0 (never) to 5 (always), and Cronbach α was .90 at T2 across groups.

Control variables included age, sex, the BMI, and previous health conditions. Age was assessed as the year of birth and recoded to age in years, while sex was assessed in 3 categories (man, woman, diverse) and used as a binary variable, as no participant indicated being diverse. Patients calculated their BMI based on their weight and height. Regarding preconditions, patients selected these from 11 prespecified health conditions (eg, cancer, cardiovascular diseases, diabetes). For the analysis, this was recoded to a binary variable (yes, no). Patients were also asked to specify whether they had any other condition that was not mentioned, which was considered for binary recoding.

### Data Analysis

All data analysis was conducted using RStudio version 4.1.2 (Posit). In the IG, the sample size was determined based on the capacity of the neurological rehabilitation clinic to conduct assessments, in addition to providing routine care to inpatients. The capacity was limited to 66 patients undergoing the assessment. To ensure that propensity score matching (PSM) could be applied, if necessary, to increase the comparability between groups without reducing the sample size further, the target sample size of the ACG was set to twice the size of the IG. For the CompG, as many participants as possible were recruited.

For the first hypothesis, the IG and ACG were combined to reach a sufficient sample size and were compared with the CompG using hierarchical-linear models (HLMs). The logistic regression to compare groups was significant for age. As there was no randomization, PSM was used to increase the groups’ comparability. Individuals were matched applying a 1:1 ratio using the *matchit* function before HLMs were carried out regarding symptom severity (including the time points T1, T2, and T4), work ability, and social participation (both measured at T2 and T4).

To test the second hypothesis, the IG and the ACG were compared over time regarding their symptom severity from T1 to T5, in addition to work ability and social participation (both measured at T2, T4, and T5) using HLMs in R. Before testing this hypothesis, a randomization check was conducted using logistic regression analysis based on the *glm* function with the dependent variable of the group. Independent variables were age, sex, the BMI, and previous health conditions. As there were no significant differences between groups at baseline (see later), HLMs were conducted to compare the IG and the ACG without further control variables. For the HLMs, data were restructured to a long format using the *gather* function, and the HLMs were tested using *lme*, allowing for random intercepts. Random slopes could not be included in the HLMs, as there were not enough observations for the HLMs to converge.

To evaluate the third and final hypothesis, 2 linear regression analyses were conducted to test whether a reduction in symptoms over time (T1-T4) predicted better work ability and social participation, respectively, at follow-up, controlled for age, sex, the BMI, and previous health conditions. To operationalize symptom reduction, a difference variable was calculated (mean symptom score at T4 – mean symptom score at T1).

## Results

### Participants

Across all groups, 1020 patients were recruited. On average, the patients’ age was 45.83 (SD 13.23) years and the BMI was 26.92 (SD 6.63). Most patients were female (763/1020, 74.8%). Many patients (669/1020, 65.6%) suffered from previous health conditions, including most commonly cardiovascular diseases (192/1020, 18.8%), obesity (157/1020, 15.4%), and mental illness (122/1020, 12.0%). Descriptive statistics for patient characteristics and more details regarding the demographic data by group are presented in Table 1.

Means (SDs) for the outcome variables are shown in Table 2.

**Table 1:** Demographic data (N=1020).

Characteristics		IG^a^ (n=73)	ACG^b^ (n=152)	CompG^c^ (n=795)
Age (years), mean (SD)		43.47 (12.65)	44.55 (11.45)	46.32 (13.59)
BMI, mean (SD)		26.73 (6.11)	27.38 (7.37)	26.76 (6.44)
**Sex, n (%)**				
	Female	49 (67.1)	112 (73.7)	602 (75.7)
	Male	24 (32.9)	39 (25.7)	160 (20.1)
	Other	0	1 (0.7)	0
**Previous conditions, n (%)**				
	Diabetes mellitus	4 (5.5)	5 (3.3)	31 (3.9)
	Cardiovascular disease	9 (12.3)	17 (11.2)	120 (15.1)
	Chronic lung disease	6 (8.2)	14 (9.2)	41 (5.2)
	Chronic kidney disease	0	1 (0.7)	5 (0.6)
	Chronic liver disease	0	1 (0.7)	6 (0.8)
	Cancer	3 (4.1)	1 (0.7)	12 (1.5)
	Autoimmune disease	9 (12.3)	12 (7.9)	69 (8.7)
	Stroke	1 (1.4)	1 (0.7)	9 (1.1)
	Obesity	8 (8.0)	24 (15.8)	79 (9.9)
	Mental illness	7 (9.6)	16 (10.5)	72 (9.1)
	Nicotine abuse	5 (6.8)	9 (5.9)	31 (3.9)

^a^IG: intervention group.

^b^ACG: active control group.

^c^CompG: comparison group.

**Table 2:** Means (SDs) for the main outcome variables over time.

Outcome variables and time points		IG^a^ (n=73)		ACG^b^ (n=152)	CompG^c^ (n=795)
**Symptom severity**					
	T1	2.73 (0.50)	2.71 (0.60)		2.67 (0.73)
	T2	2.37 (0.54)	2.35 (0.56)		2.46 (0.62)
	T3	2.29 (0.51)	2.32 (0.57)		N/A^d^
	T4	2.22 (0.57)	2.20 (0.60)		2.45 (0.67)
	T5	2.06 (0.56)	2.18 (0.65)		N/A
**Work ability**					
	T2	5.13 (2.56)	4.97 (2.21)		4.46 (2.65)
	T4	5.43 (2.40)	5.65 (2.51)		4.72 (2.89)
	T5	5.69 (2.87)	5.65 (2.77)		N/A
**Social participation**					
	T2	3.34 (0.88)	3.43 (0.76)		3.20 (0.84)
	T4	3.34 (0.89)	3.53 (0.92)		3.39 (0.86)
	T5	3.67 (0.90)	3.46 (0.95)		N/A

^a^IG: intervention group.

^b^ACG: active control group.

^c^CompG: comparison group.

^d^N/A: not applicable.

### Testing Hypothesis 1: Intervention Effects Between the IG and the ACG vs the CompG

Logistic regression showed a significant association between age and group (β=–.04, *P*<.001; see Table 3). Thus, PSM was applied to increase the comparability between groups, resulting in 137 matched pairs.

Comparing symptom severity across the time points T1, T2, and T4, we found a significant reduction in both the IG and the ACG over time (T1 vs T2: β=.13, *t*_549_=5.67, *P*<.001; T2 vs T4: β=.06, *t*_549_=2.83, *P*=.01). The main effect of the group was significant (β=–.15, *t*_549_=–2.65, *P*=.01), and the reduction in symptoms over time was more pronounced in the IG and ACG compared to the CompG (T1 vs T2 between groups: β=.14, *t*_549_=4.31, *P*<.001; T2 vs T4 between groups: β=.14, *t*_549_=4.57, *P*<.001).

**Figure 2:**
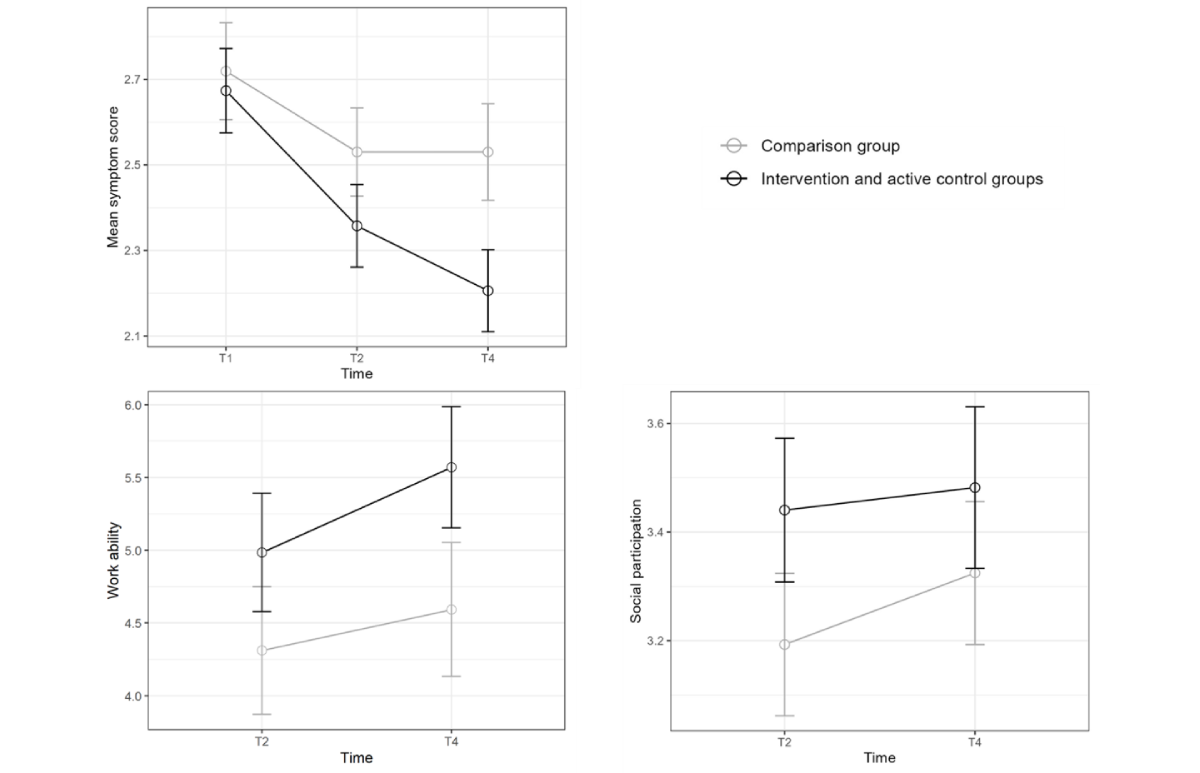
Symptom development, work ability, and social participation over time in the IG, ACG, and CompG. Error bars represent SDs in the respective groups. ACG: active control group; CompG: comparison group; IG: intervention group.

For work ability, there was no significant difference between T2 and T4, but the work ability was higher in the IG and ACG compared to the CompG (β=.78, *t*_271_=2.73, *P*=.01). Finally, social participation increased over time (β=.13, *t*_277_=1.98, *P*=.049) and was higher in the IG and ACG (β=.27, *t*_277_=2.89, *P*=.004), but there was no significant interaction effect. Figure 2 shows the development of symptom severity, work ability, and social participation over time. All statistics are reported in Table 4.

**Table 3:** Randomization check for the 2 treatment groups (IG^a^ and ACG^b^) vs the CompG^c^.

Characteristics	Estimate	SE	*z* Value	*P* value
(Intercept)	0.60	0.61	0.97	.332
Sex	0.45	0.25	1.85	.065
Age	–0.04	0.01	–4.69	<.001
Precondition	0.06	0.24	0.24	.812
BMI	0.02	0.02	1.07	.286

^a^IG: intervention group.

^b^ACG: active control group.

^c^CompG: comparison group.

**Table 4:** HLM^a^ results for the comparison between the treatment groups (IG^b^ and ACG^c^) and the CompG^d^.

Outcome variables			Estimate		SE		*t* (*df*)		*P* value
**Symptom severity**									
	(Intercept)	2.58		0.04		57.87 (549)		<.001	
	Time point T1/T2	0.13		0.02		5.67 (549)		<.001	
	Time point T2/T4	0.06		0.02		2.84 (549)		.005	
	Group	–0.15		0.06		–2.65 (549)		.008	
	Time point T1/T2 × group	0.14		0.03		4.31 (549)		<.001	
	Time point T2/T4 × group	0.14		0.03		4.57 (549)		<.001	
**Work ability**									
	(Intercept)	4.25		0.21		20.10 (271)		<.001	
	Time point T2/T4	0.28		0.15		1.87 (271)		.063	
	Group	0.78		0.29		2.73 (271)		.007	
	Time point T2/T4 × group	0.30		0.21		1.43 (271)		.155	
**Social participation**									
	(Intercept)	3.18		0.07		46.70 (277)		<.001	
	Time point T2/T4	0.13		0.07		1.98 (277)		.049	
	Group	0.27		0.09		2.89 (277)		.004	
	Time point T2/T4 × group	–0.09		0.09		–0.96 (277)		.339	

^a^HLM: hierarchical-linear model.

^b^IG: intervention group.

^c^ACG: active control group.

^d^CompG: comparison group.

### Testing Hypothesis 2: Intervention Effects Between the IG and the ACG

Logistic regression did not reveal any significant associations between sociodemographic variables and group allocation (Table 5). Hence, no PSM was used prior to comparing the IG and the ACG regarding the effectiveness of the assessment, but group differences were directly compared.

Concerning symptom severity, the HLM revealed a decrease over time in both the IG and the ACG but no group differences between the IG and the ACG. All time points significantly differed from the first time point (T1 vs T2: β=.27, *t*_456_=8.29, *P*<.001; T2 vs T3: β=.33, *t*_456_=8.10, *P*<.001; T3 vs T4: β=.32, *t*_456_=7.87, *P*<.001; T4 vs T5: β=.18, *t*_456_=5.32, *P*<.001).

**Figure 3:**
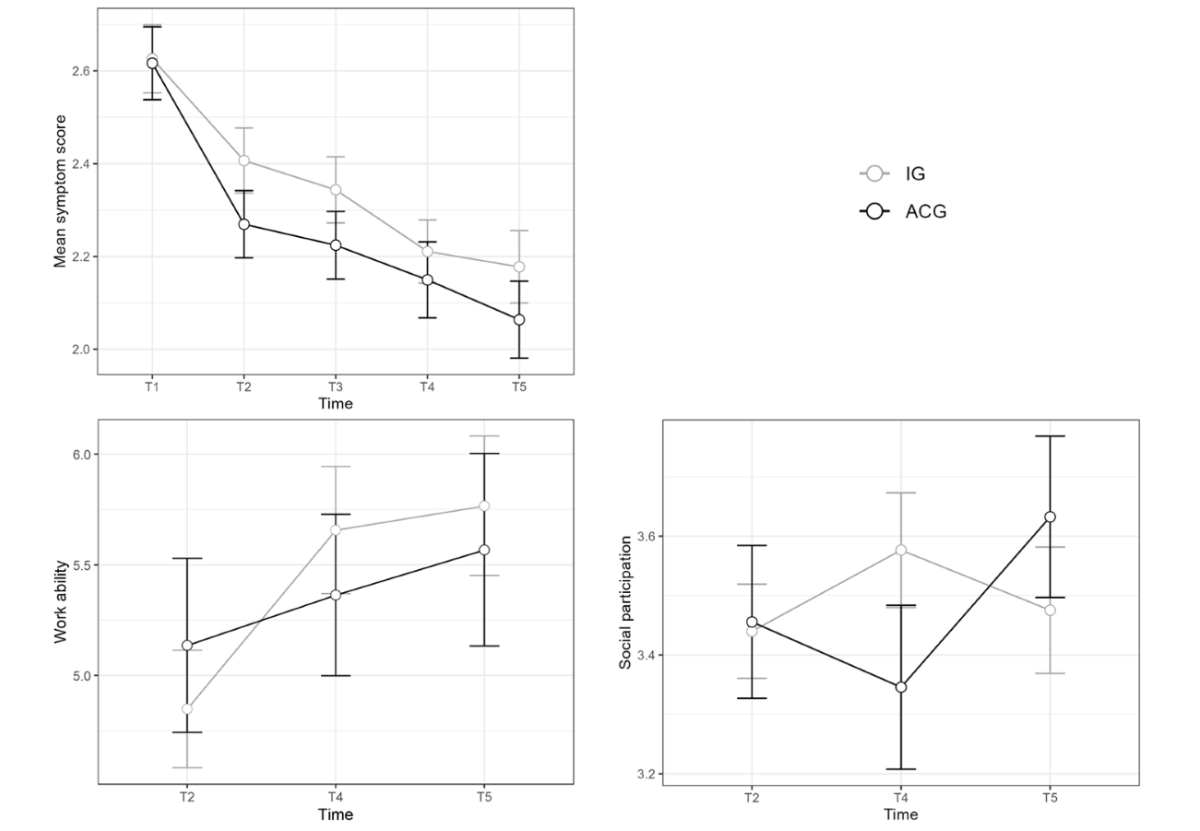
Symptom development, work ability, and social participation over time in the IG and the ACG. Error bars represent 1 SD in the respective groups. Treatments for the IG included diagnostic assessment, personal support, and digital interventions, while those for the ACG included only personal support and digital interventions. ACG: active control group; IG: intervention group.

The work ability of patients increased over time (T2 vs T4: β=–.58, *t*_230_=–4.25, *P*<.001; T4 vs T5: β=–.34, *t*_230_=–2.59, *P*=.01), but no difference between the IG and the ACG was found. There were also no significant differences in social participation over time or between groups. Figure 3 shows the development of symptom severity, work ability, and social participation over time. All inference statistics are reported in Table 6.

**Table 5:** Randomization check for the IG^a^ and ACG^b^.

Characteristics	Estimate	SE	*z* Value	*P* value
(Intercept)	–0.26	0.92	–0.28	.780
Sex	0.32	0.35	0.92	.360
Age	–0.01	0.01	–0.55	.581
Precondition	0.15	0.36	0.43	.666
BMI	–0.02	0.03	–0.64	.520

^a^IG: intervention group.

^b^ACG: active control group.

**Table 6:** HLM^a^ results for the comparison between the IG^b^ and the ACG^c^.

Outcome variables		Estimate	SE	*t* (*df*)	*P* value	
**Symptom severity**						
	(Intercept)	2.35	0.06	39.38 (456)	<.001	
	Time T1/T2	0.27	0.03	8.29 (456)	<.001	
	Time T2/T3	0.33	0.04	8.10 (456)	<.001	
	Time T3/T4	0.32	0.04	7.87 (456)	<.001	
	Time T4/T5	0.18	0.03	5.32 (456)	<.001	
	Group	–0.09	0.10	–0.91 (114)	.365	
	Time T1/T2 × group	0.09	0.05	1.47	.143	
	Time T2/T3 × group	0.03	0.07	0.45 (456)	.653	
	Time T3/T4 × group	0.00	0.07	–0.02 (456)	.982	
	Time T4/T5 × group	0.03	0.05	0.48 (456)	.632	
**Work ability**						
	(Intercept)	5.42	0.27	20.34 (230)	<.001	
	Time T2/T4	–0.58	0.13	–4.25 (230)	<.001	
	Time T4/T5	–0.34	0.13	–2.59 (230)	.010	
	Group	–0.07	0.43	–0.16 (115)	.875	
	Time T2/T4 × group	0.36	0.22	1.65 (230)	.101	
	Time T4/T5 × group	0.13	0.22	0.60 (230)	.547	
**Social participation**						
	(Intercept)	3.50	0.07	46.82 (232)	<.001	
	Time T2/T4	–0.06	0.06	–0.90 (232)	.368	
	Time T4/T5	0.02	0.06	0.34 (232)	.730	
	Group	–0.02	0.12	–0.16 (116)	.875	
	Time T2/T4 × group	0.03	0.10	0.34 (232)	.737	
	Time T4/T5 × group	–0.18	0.10	–1.70 (232)	.091	

^a^HLM: hierarchical-linear model.

^b^IG: intervention group.

^c^ACG: active control group.

### Testing Hypothesis 3: Association Between Symptom Reduction, Work Ability, and Social Participation

Two linear regression analyses were conducted to test whether a reduction in symptoms between T1 and T4 had positive effects on patients’ work ability and social participation. The analyses revealed that a greater reduction in symptoms does not predict a higher reported work ability when controlling for age, sex, the BMI, and previous health conditions (β=–.18, *t*_111_=–1.92, *P*=.06; R^2^=0.32) but does predict a higher social participation (β=–.34, *t*_111_=–2.11, *P*=.04, R^2^=0.24). All statistics are reported in Multimedia Appendix 2 (Tables A2 and A3).

## Discussion

### Principal Findings

The aim of this study was to develop and evaluate an interdisciplinary health care facilitation program based on professional support and digital interventions by increasing the self-management of patients with PACS. Within the ASAP project, the IG received a diagnostic assessment in a clinic, accordingly tailored digital interventions, and support from a human personal pilot. The ACG received the digital interventions and personal support but no assessment and no interventions tailored to the assessment results.

Our first hypothesis was that both groups receiving interventions would benefit when compared to the passive CompG. Regarding symptom severity, there was a larger reduction in both the IG and the ACG compared to the CompG. Furthermore, work ability and social participation were higher in both treatment groups (IG and ACG). Our second hypothesis was that patients in the IG would benefit more than patients in the ACG as they received a diagnostic assessment that could potentially lead to more tailored treatment in the long run and also received a more tailored digital intervention program than patients in the ACG. However, based on the inferential statistics, this hypothesis failed to hold true. Both groups showed a similar level of symptom reduction and an increase in their work ability over time. Third, we hypothesized that patients’ symptom reduction over the course of the intervention program would predict their work ability and social participation at follow-up. This was only the case for social participation but not work ability as the *P* value just failed to reach <.05.

It is common in PACS that symptoms change and decrease over time without any intervention [37]. Nevertheless, a substantial number of patients still suffer from long-term consequences of the infection [3], indicating that prevention and tailored interventions are needed to manage the impact on individuals and society. Promisingly, there were positive effects in the treatment groups compared to the CompG, showing that a combination of support from a personal pilot over the phone and digital rehabilitation treatment can help facilitate recovery (see also Ref. [21]). However, due to the study design, it is not possible to disentangle the specific effects of either intervention on its own (ie, whether the personal support or digital interventions were more effective in symptom reduction). Hence, more studies are needed to find out whether and how specific tailored support to facilitate the treatment process, as well as digital interventions, can help patients with PACS recover.

The specific intervention approaches included support from personal pilots, who helped manage the health care system via regular, scheduled phone calls and on-demand meetings. The personal pilots helped the patients manage 3 different challenges: their daily life with their symptoms, the difficult process of finding individual diagnostic and treatment options, and taking part in a study. Although the personal pilots were not all trained as therapists, they provided vital support that was recognized by the patients, who perceived them as important partners in their recovery process. Earlier studies have also found that a supportive relationship and therapeutic companionship can be established through online platforms [38,39]. However, some interventions may require more time to achieve similar outcomes as face-to-face settings [40]. Fortunately, the ASAP project, which ran for approximately 10 weeks, was able to maintain regular contact and overcome this potential challenge.

Another intervention in this study was the use of digital interventions through a digital rehabilitation platform, including physical exercises, mindfulness training, and sensory as well as functional training. This intervention was delivered without a physical therapist. In general, patients describe web-based interventions as useful and satisfactory when they are delivered by a therapist [41], and engage more in live or synchronous digital interventions [42]. In the ASAP project, interventions were asynchronous, which had the advantage that patients could complete the program more flexibly and integrate the interventions in their daily routines and physical conditions, as well as adapt them to their current symptoms. Additionally, personal pilots were available if patients needed any kind of support, for example, for technical support or if exercises did not fit their demands.

Based on the feedback of patients as well as earlier research [41,42], it can be suggested that digital interventions and personal support should be combined. This was the case in the ASAP project, and the combination of both digital therapy and personal support could account for positive effects. These positive effects also occurred after the digital interventions were finished over a time frame of another 6 weeks, showing the sustainability of the health care facilitation program concerning symptoms. The sustainable reduction in symptoms and even further improvements in the intervention and ACGs indicated that patients might have learned skills and active coping strategies that they continued to use and transferred to other life areas, in addition to finding good treatment options within the health care system. Importantly, our results are in line with a review published in *Lancet Respiratory Medicine* that emphasized that the complex and multifactorial impairments of patients with PACS need a coordinated, multidisciplinary approach, including support and behavioral interventions, to develop coping and compensatory strategies [43]. The ASAP project has the advantage that the personal pilots can care for more patients in a flexible, individual approach.

Despite the likelihood that the interventions introduced in this study had positive effects on the patients’ symptoms, these effects in self-reported symptom severity could also be attributed (partly) to the placebo effect [44,45]. As can be seen in Figure 2, the symptoms in the IG and ACG already decreased between T1 and T2, where no specific intervention targeting the management of symptoms was offered. This could be attributed to unspecific interventions, such as general information about hygiene, nutrition, and exercise, and thus the placebo effect. Additionally, other psychological mechanisms, such as social desirability or the new relationship with the pilot, could account for the positive effects of the intervention. Nevertheless, there was an additional decrease in symptom severity when specific interventions were introduced, so it is unclear at this point how much of the decrease was caused by the placebo effect and specific intervention effects. It should thus be established in future research how important the placebo effect is in the reduction of PACS symptoms and how it can be effectively used to strengthen intervention approaches.

The results regarding social participation and work ability showed a different pattern. Although the interaction term indicating group differences between the treatment groups and the CompG was significant for symptom reduction, there was no such advantage of the treatment regarding these constructs that are crucial for the quality of life of patients with PACS [46]. It might be that the project was too focused on facilitating health care for symptoms, understanding these as major barriers to work ability and social participation and not focusing enough on the quality of life. Nevertheless, a reduction in symptoms over the course of the project predicted higher social participation at follow-up, indicating that the interventions with the final goal of symptom reduction might still have positive effects on the quality of life via indirect mechanisms. Dahmen et al [45] also found that there is no difference regarding the outcome of social participation, depending on whether rehabilitation patients receive digital or in-person aftercare.

Concerning work ability, Zwerenz et al [46] found positive effects of psychological aftercare through web-based programs targeting not only the patient but also the workplace. Although the personal pilots had the goal to facilitate coping with symptoms in daily life and thus increase social participation, it seems that despite a reduction in PACS symptoms related to social participation and thus the quality of life, work ability is determined by a number of other factors [47]. This could be explained by the fact that work ability and social participation not only concern the individual patient but also the broader social network (ie, families and workplaces). Hence, the ASAP project might need to be broadened to also target the patients’ environment to improve aspects regarding the quality of life.

Finally, the 2 treatment groups did not differ from each other over time concerning any outcome measure. This would suggest that the 3-day diagnostic assessment seems to have failed to create long-term advantages in the IG despite a comprehensive diagnostic program assessing physical, respiratory, neurological, and neuropsychological functions. The ACG consisted of shared decision-making of the personal pilots, together with patients, regarding digital interventions focusing on fatigue, neurocognitive symptoms, or cardiorespiratory symptoms. This approach managed to guide individualized digital treatment decisions in an easy and cost-effective way, so no advantages of the IG with digital interventions tailored to the diagnostic assessment could be detected. Nevertheless, there are several reasons to assume that the clinical assessment could have positive effects on symptom reduction and social participation in the long run: Patients especially valued the comprehensive (neuro)psychological diagnostics and the associated contact with a therapist. A lot of patients with PACS disclosed that they had not felt understood and validated in their symptoms and concerns before [48]. The patients who received the 3-day diagnostic assessment provided positive feedback regarding the time and care that went into their assessment. The clinical assessment was particularly designed to provide patients with objective findings that could guide further outpatient specialty and rehabilitative care. However, finding and completing specialist and rehabilitative care that meets individual needs might take a long time and the effects, accordingly, were thus not captured in follow-up measures of this study [49].

After the assessment, patients were followed up over the course of approximately 3 months. Despite clear recommendations for medical rehabilitation provided in the diagnostic assessment, there are several health care–associated challenges as well as personal barriers that were not sufficiently considered in this study. Thus, the uptake or completion of a subsequent rehabilitative intervention might not have been possible during the time frame that was covered by the study’s outcome assessments. Another study demonstrated that 80% of all rehabilitation patients return to work within 2 years postrehabilitation, indicating a positive effect of rehabilitation tailored to individual needs. Nevertheless, further studies need to clarify whether this is also the case for patients with PACS [50]. It seems likely that a comprehensive assessment can be used to reliably determine the need for therapy as well as rehabilitation but that barriers in the health care system prevent patients from taking up effective rehabilitation treatment, as has been identified in other conditions [51,52].

### Limitations and Recommendations for Future Research and Practice

Although this study used the gold standard with a randomized controlled trial between the IG and the ACG, it should be kept in mind that the CompG was only matched but not randomized. The reason was that there were ethical concerns to not administer the intervention to patients with PACS in the same recruitment in which other patients received the intervention. It was anticipated that many of the potential patients with PACS would have already sought help or treatment, and we did not want to cause substantial frustration and despair when randomizing them to a passive control group. Due to the different recruitment pathways, patients could be immediately informed that they would only receive questionnaires. To statistically control for differences between groups, we chose the PSM approach.

The inclusion criteria were largely the same for all groups, including patients who had the same symptoms of PACS, were between 18 and 60 years old, had not received previous treatment, and did not work in the health care sector. Nevertheless, patients in the CompG were recruited from the whole country of Germany, not just Bavaria, to reach a potentially larger pool of participants. In the future, randomization and completely similar inclusion criteria should be applied to allocate patients to all groups, including a passive control group. This would be beneficial to ensure the comparability of patients across groups as well as the generalizability of findings.

In this study, the follow-up period after completing the digital assessments was only 6 weeks due to the total funding period of 12 months. Despite the apparently stable improvements in both the IG and the ACG, this period is too short to conclude that the program caused sustainable improvements. This is especially true due to the dynamic and fluctuating nature of PACS symptoms that many patients experience. Furthermore, no effects of the diagnostic assessment were found, which might have been, among other reasons, because of a too short follow-up period. Patients received recommendations for future interventions, such as rehabilitation after the diagnostic assessment, which was approximately 12 weeks before the follow-up measure. During that time, most patients could not apply for and complete rehabilitation, so the long-term effects of the diagnostic assessment were potentially underestimated. Despite the limited follow-up period that should be addressed in future intervention evaluations, we believe that as one of the first studies examining an intervention aimed at treating PACS, our research provides valuable insights into potential treatment approaches during this pressing health care challenge.

In addition, digital interventions and the support of personal pilots could not be evaluated independently in this design, but the results indicated that a combined approach is effective in treating PACS. It is possible that the patients took part in other interventions, as the personal pilots empowered them to find effective medical treatment. This can also be seen as a strength of this evaluation as it examines an applied approach in a realistic health care setting. However, it is possible that some of the effects that we found were caused by the placebo effect rather than specific intervention effects, as indicated in the discussion. In this study, we did not have a placebo control arm, which would be beneficial for future research. This group could include unspecific interventions (eg, information about the COVID-19 pandemic and PACS symptoms) as well as counseling that is not focused on symptom management).

Finally, all outcome assessments were taken at a subjective level only and need to be validated with objective measurements. This is also true for the condition of PACS, as only patients in the IG and ACG were assessed for whether they had PACS. More objective assessments should be applied to carefully diagnose PACS and and improvements in the condition (eg, spirometry or electrocardiography).

### Conclusion

Future research should apply study designs with more standardized recruitment, rigorous randomization, objective measures, and longer follow-up periods to strengthen the validity and generalizability of results. Nevertheless, this study clearly demonstrated the potential and effectiveness of empowering patients to cope with their symptoms. Therefore, professional support should be incorporated in medical treatment as it seems to be an effective way to support patients with PACS. Providing patients with human support and thereby also knowledge about their condition, possible treatment options, and symptom management can help them cope with their situation. In the shared decision-making approach in the ACG, the digital interventions were individualized by involving patients, which resulted in symptom reduction when compared to the passive CompG. However, this group was recruited via a different recruitment pathway, which should be considered when interpreting the findings. Encouraging patients to play an active role in their own care can help them feel empowered. This can include strategies of developing a self-care plan, tracking symptoms, and monitoring medication use, as well as completing their exercises in the digital interventions. Promising digital interventions are breathing and relaxation exercises, mindfulness training, meditation, physical strength tasks, and sensory as well as functional training. Improving patients’ access to care and their capability to take an active part in their treatment can potentially help reduce symptoms and thus alleviate the pandemic burden on the society.

## Appendix

Multimedia Appendix 1 CONSORT-eHEALTH checklist (V 1.6.1).

Multimedia Appendix 2 Supplementary Tables A1-A3.

## Abbreviations

ACG: active control group
ASAP: Assisted Immediate Augmented Post-/Long-COVID Plan
CompG: comparison group
GP: general practitioner
HLM: hierarchical-linear model
IG: intervention group
PACS: postacute COVID-19 syndrome
PSM: propensity score matching

## Data Availability

The data and syntax for this manuscript are available in Ref. [53].
