# Supplementary material for: Longitudinal Evaluation of an Integrated Post–COVID-19/Long COVID Management Program Consisting of Digital Interventions and Personal Support: Randomized Controlled Trial
Source: J Med Internet Res. 2023 Oct 4;25:e49342. doi: 10.2196/49342 (PMC10563866; doi:10.2196/49342)
Supplement: Multimedia Appendix 2 [file jmir_v25i1e49342_app2.docx]

**Multimedia Appendix**

**Table A1**

*Diagnostic tests in the assessment.*

| **Domain** | **Tests** |
| --- | --- |
| Physical conditions | Height, weight, pulse, electrocardiography, oxygen saturation, body temperature, blood pressure, breathing frequency |
| Respiratory functions | Spirometry |
| Neurological functions | EEG, Electroneurography (ENG), initial neurological examination, evoked potentials |
| (Neuro-)Psychological functions | Attention test battery, alertness test, California Verbal Learning test, Five Point test, visual scanning test, working memory test |
| Stress test | Schellong orthostasis test, ergometry |
| Taste and smell | Taste and smell test (SS-16) |
| Workability | Occupational assessment |
| Internal medicine | Echography, carotis-duplex sonography |

**Table A2**

*Linear regression analysis for the effects of symptom development on workability.*

|  | Estimate | *SE* | *β* | *T* | *P* |
| --- | --- | --- | --- | --- | --- |
| (Intercept) | 7.04 | 1.49 |  | 4.73 | <.001 |
| Symptom development (T4-T1) | -0.92 | 0.48 | -.18 | -1.92 | .06 |
| Sex | -0.77 | 0.54 | -.13 | -1.43 | .16 |
| Age | -0.04 | 0.02 | -.16 | -1.66 | .10 |
| Previous health conditions | -0.45 | 0.56 | -.08 | -0.81 | .42 |
| BMI | 0.04 | 0.04 | .10 | 1.06 | .29 |

*Note*: R^2^=.102

**Table A3**

*Linear regression analysis for the effects of symptom development on social participation.*

|  | Estimate | *SE* | *β* | *T* | *P* |
| --- | --- | --- | --- | --- | --- |
| (Intercept) | 3.01 | 0.50 |  | 6.02 | <.001 |
| Symptom development (T4-T1) | -0.34 | 0.16 | -.20 | -2.11 | .04 |
| Sex | 0.03 | 0.18 | .01 | 0.14 | .89 |
| Age | 0.00 | 0.01 | .003 | 0.03 | .98 |
| Previous health conditions | -0.20 | 0.19 | -.11 | -1.06 | .29 |
| BMI | 0.02 | 0.01 | .13 | 1.33 | .19 |

*Note*: R^2^=.102
